# A two-step machine learning approach for predictive maintenance and anomaly detection in environmental sensor systems

**DOI:** 10.1016/j.mex.2025.103181

**Published:** 2025-01-28

**Authors:** Saiprasad Potharaju, Ravi Kumar Tirandasu, Swapnali N. Tambe, Devyani Bhamare Jadhav, Dudla Anil Kumar, Shanmuk Srinivas Amiripalli

**Affiliations:** a Department of CSE, Symbiosis Institute of Technology, Symbiosis International (Deemed University), Pune, India; b Department of CSE, Koneru Lakshmaiah Education Foundation, Vaddeswaram, Andhra Pradesh, India; c Department of Information Technology, K. K.Wagh Institute of Engineering Education & Research, Nashik, Maharashtra, India; d AIML Department, Sanjivani University, Kopargaon, Maharashtra, India; e Department of CSE, Lakireddy Bali Reddy College of Engineering, NTR District, Andhra Pradesh, India; f Department of CSE, GST, GITAM University, Visakhapatnam, Andhra Pradesh, India

**Keywords:** Environmental sensor systems, Anomaly detection, Predictive maintenance, Supervised learning, Unsupervised learning, Integration of Unsupervised and Supervised learning

## Abstract

Environmental sensor systems are essential for monitoring infrastructure and environmental quality but are prone to unreliability caused by sensor faults and environmental anomalies. Using Environmental Sensor Telemetry Data, this study introduces a novel methodology that combines unsupervised and supervised machine learning approaches to detect anomalies and predict sensor failures. The dataset consisted of sensor readings such as temperature, humidity, CO, LPG, and smoke, with no class labels available. This research is novel in seamlessly blending unsupervised anomaly detection using Isolation Forest to create labels for data points that were previously unlabeled. Finally, these generated labels were used to train the supervised learning models such as Random Forest, Neural Network (MLP Classifier), and AdaBoost to predict anomalies in new sensor data as soon as it gets recorded. The models confirmed the proposed framework's accuracy, whereas Random Forest 99.93 %, Neural Network 99.05 %, and AdaBoost 98.04 % validated the effectiveness of the suggested framework. Such an approach addresses a critical gap, transforming raw, unlabeled IoT sensor data into actionable insights for predictive maintenance. This methodology provides a scalable and robust real-time anomaly detection and sensor fault prediction methodology that greatly enhances the reliability of the environmental monitoring systems and advances the intelligent infrastructure management.

• Combines Isolation Forest for anomaly labeling and supervised models for anomaly prediction.
• Scalable and adaptable for diverse IoT applications for environmental monitoring.
• Provides actionable insights through anomaly visualization, revealing patterns in sensor performance.

## Graphical abstract

**Figure:**
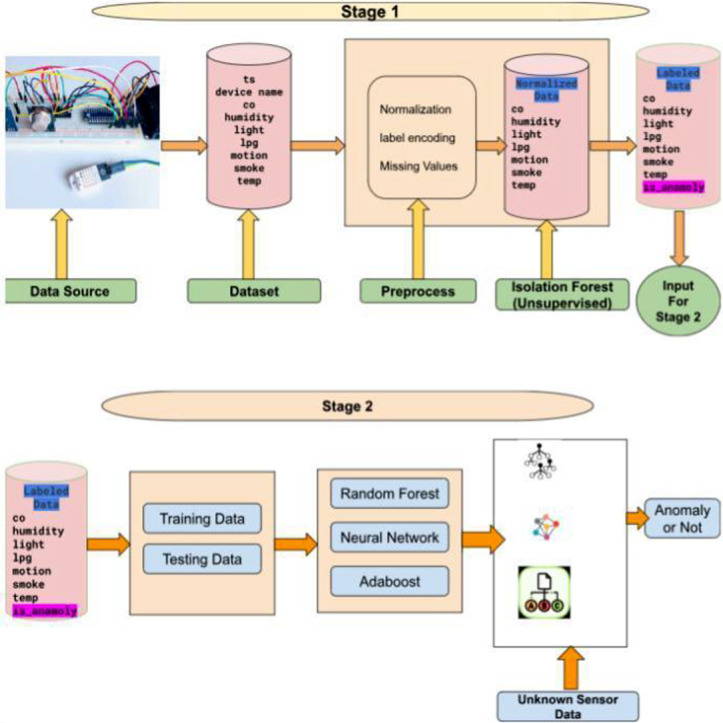

Specifications table

**Table:** 

Subject area:	Computer Science
More specific subject area:	*Machine learning*
Name of your method:	Integration of Unsupervised and Supervised learning
Name and reference of original method:	–
Resource availability:	https://www.kaggle.com/datasets/garystafford/environmental-sensor-data-132k

## Background

Environmental sensor systems are becoming a necessary part of the infrastructure and condition monitoring, providing crucial data for decisions in many domains, including smart cities, industrial automation and environmental protection as well as being essential for improving the fitness, health and independence of all populations of all ages [1]. Most of these systems have several sensors but some detect temperature and humidity while others track carbon monoxide (CO), liquid petroleum gas (LPG), smoke levels and motion. However, the reliability and accuracy of data from such systems are foundational to the safe and efficient operation and decisions which will be made as such systems expand. These high reliability systems are increasingly important but reduced to faults and work below sensor fault and environment anomaly induced high reliability standards [2]. All of which can lead to slow response times, or even critical failures in real-time systems. Consequently, it is necessary to find anomalies in sensor readings, and to be able to predict ahead of time when these sensors will fail. These issues are more important when environmental sensor systems are used as the backbone of critical applications. Each example underscores the importance of efficient management in ensuring the reliability and accuracy of sensor systems, and in smart cities, sensors monitor air quality, traffic flow, integrity of the infrastructure. They could be providing poor information which can result in poor decisions, since these sensors have the potential to fail to provide the information they intended to provide. The type of failure can be very important in industrial automation, where sensors control machinery and safety systems: They can fail and cost expensive downtimes or even dangerous conditions [3]. Similarly, sensor data are routinely employed to gauge ecological parameters such as water level, temperature, and concentration of pollutants in the service of environmental conservation and protection. If these systems make mistakes, they can hamper conservation techniques or hold up responses to environmental threats. These examples show why we need solutions to identify and resolve anomalies in sensor data before they turn into major issues.

One of the fundamental challenges environmental sensor systems face is the absence of labeled datasets, which are essential for training machine learning models to detect anomalies and predict faults. In contrast to traditional supervised learning problems, where labeled data underlies the building of models, sensor data in real-world tasks will typically lack predefined labels. First, labeling large quantities of sensor data is time-consuming and expensive, requiring domain knowledge and man hours. In addition, anomalies are rare and diverse, inherently difficult to categorize and label exhaustively. Since in this approach, there exists a considerable gap in using machine learning algorithms that usually require labeled datasets for training and validation, and our main focus is to strive to close this sufficiently [4].

The variability of sensor anomalies is another complicating factor. Such anomalies often result from various source types: hardware degradation, external interferences, or environmental change. For example, if these sensors are used for a particular purpose like a temperature sensor then they could record its unusual spikes from around outside heating or a CO sensor could fail due to hardware wear and tear [5]. These anomalies must be realized across modes of sensor deployment and across types of sensors, and are dependent on knowledge about the normal operating space across which we would experience such anomalies, as well as knowledge about possible failure modes, always. The variability of the anomaly sources further complicates developing robust models for anomaly detection.

Since such errors can cascade all the way to downstream applications without appropriate anomaly detection. Errors are exacerbated by automated decision-making systems due to incorrect sensor readings [6,7]. In an industrial setting for instance, if you're not getting accurate humidity readings, you will have incorrect climate control system adjustments that consume energy and resources that might be better spent elsewhere [8]. Motion sensor failure in a traffic management system can misrepresent vehicle flow making signal timings inferior to what they might be. Broad implications of sensor anomalies are presented, in terms of how they affect the data accuracy represented as captured and the effectiveness and reliability of the systems that the said sensors service [9].

This problem gives rise to several key research questions, such as identifying effective methods to exploit unlabeled sensor data for anomaly detection and determining strategies to transition from unsupervised anomaly detection to supervised predictive modeling. First, we seek to understand how to design a scalable solution to operate in real-time environments and deal with the diversity of sensor faults. How can such a solution be validated to ensure it's reliable in real-world situations? The resulting need is to find a systematic approach that not only detects anomalies in an unlabeled data set but also utilizes these anomalies to predict anomalies in future sensor readings.

Several key objectives must be addressed for the expected solution to this problem. Second, it must be able to process large amounts of unlabeled sensor data to autonomously find anomalies in an unsupervised manner. We use the Isolation Forest algorithm, a robust unsupervised learning approach to isolate outliers by random partitions to achieve the above. This application favors a method that can achieve good results on large datasets with few computational overheads, and such a method is the case. Second, a mechanism to turn an unlabeled dataset into a labeled format should be configured. The Isolation Forest produces anomaly labels which, when fed into a supervised learning algorithm, successfully bridge the gap between raw data and predictive modeling.

Next, supervised machine learning algorithms train these predictive models, which predict anomalies in future sensor measurements [10]. We evaluate the effectiveness of different algorithms in answering this question with Random Forest, Neural Network (MLPClassifier) and AdaBoost. Each model brings unique strengths: They are good at handling noisy high dimensional data (Random Forest), at seeing complex, highly nonlinear patterns (Neural Networks), and in improving upon your predictions by iterating through tricky, hard-to-classify instances (AdaBoost). Fourth, we validate the solution with accuracy, precision, recall, and F1 score to show that the solution is robust. They serve as a comprehensive evaluation of the models’ ability to separate normal and anomalous sensor readings.

Finally, the methodology is designed to be scalable to other sensor systems and environments. Thus, the proposed solution is not coupled to any particular sensor types or configurations. However, considering the number of use cases that it can be applied to, it can provide us with a flexible framework that can be designed and implemented to meet other use cases, e.g., industrial automation or environmental monitoring. To fill this gap, a methodology that combines unsupervised and supervised learning is proposed to convert raw data to actionable insight, resulting in a robust framework for real time anomaly detection and prediction maintenance in environmental sensor systems.

A variety of other domains will be impacted by this research. In smart cities the methodology can be applied to improve the reliability of systems monitoring air quality, traffic flow, and infrastructure health in detecting anomalies quickly enough to prevent compromising public safety or efficiency. The proposed approach enables proactive maintenance in industrial settings and decreases costs of downtime and maintenance needed to make the system operational. This improved monitoring accuracy increases environmental conservation efforts by improving the speed of responding to threats to ecology. The scalability of the methodology allows us to bring it to other environments and become a good solution for IoT based monitoring.

Before proposing the proposed flow, some of the existing literature survey in which unsupervised methods are applied when there is no target variable for various fields in Table 1.

**Table 1:** Summary of existing survey.

Approach	Objectives	Challenges
Hybrid machine learning ensemble (LOF, One-Class SVM, Autoencoder) [11]	Real-time anomaly detection in Industry 4.0 systems, improving reliability and operational efficiency	Handling unlabeled data, achieving robust and scalable anomaly detection
Hybrid deep learning (CNN, GRU, Bi-LSTM) - DeepDetect [12]	Detecting DoS and R2L threats, improving IoT network security and reducing false alarms	Overcoming poor positive and detection rates, handling unlabeled attack types
One-class SVMs on industrial data [13]	Predictive maintenance, anomaly detection in real-world manufacturing, improving recall and reliability	Training with steady-state data, addressing scalability and adaptability
Hybrid deep learning (multi-channel CNN, LSTM) [14]	Unsupervised anomaly detection in spatiotemporal data	Handling spatial-temporal dependencies without labels, improving baseline accuracy
Systematic review (44 studies on fault detection) [15]	Understanding scalability, accuracy, and adaptability of ML methods in predictive maintenance	Concept drift, nonstationary processes, lack of labeled data
HTM-based fault detection and recovery [16]	Detecting and recovering IoV faults using injected labels and nearby vehicle data	Handling unlabeled faults, noise resistance, and achieving high accuracy
Systematic review of IoT anomaly detection methods (64 papers) [17]	Categorizing methods and applications, addressing scalability and real-time requirements	Lack of labels, concept drift, multi-sensor integration
Elliptical Summaries Anomaly Detection (ESAD) [18]	Anomaly detection in marine ecosystems using clustering and visualization techniques	Handling high-dimensional, unlabeled environmental data
Gaussian Mixture Model (GMM) for IoT temperature monitoring [19]	Preventive maintenance and operational efficiency in IoT	Real-time requirements, resource efficiency, and handling unlabeled data
Hybrid ML framework (HMM and SVM) for healthcare IoT [20]	Real-time anomaly detection and improving IoT device security	Data inconsistency, lack of labels, low-resource integration
Systematic mapping study on industrial machinery anomaly detection (IoT and ML) [21]	Enhancing AD efficiency and scalability for predictive maintenance in Industry 4.0	Limited fault data, system integration, edge-compatible algorithms
IoT cybersecurity ensemble ML framework with Bayesian optimization [22]	Real-time anomaly detection, robustness and scalability in heterogeneous IoT	High-dimensional data, hyperparameter sensitivity, data heterogeneity
Hybrid ML and DL approach (IoT 23 dataset) [23]	Flexible and efficient anomaly detection balancing accuracy and cost	Resource constraints, preprocessing heterogeneity, scalability
Review of Smart Environment Monitoring (SEM) systems [24]	Enhancing sustainability and environmental health through robust SEM systems	Sensor interoperability, noisy data, limited sample sizes
Review of IoT anomaly detection techniques [25]	Improving scalability, security, and interpretability of IoT systems	Data heterogeneity, scalability, lack of labeled data
Active learning (AL) for environmental monitoring [26]	Streamlining anomaly detection in high-dimensional sensor data	Seasonality, non-stationarity, nonlinear dynamics
State-of-the-art review on predictive maintenance in industrial systems [27]	Enhancing robustness, scalability, and interpretability of ML methods	High-dimensional noisy data, concept drift, labeled data scarcity
ML for IoT-based vertical plant walls [28]	Effective anomaly detection for improved indoor climate management	Complex patterns, lack of labeled data, manual anomaly generation
Traffic anomaly detection using DAD dataset [29]	Establishing a benchmark for IoT traffic analysis using ML	Unbalanced data, feature engineering, scalability
Anomaly detection in IoT networks using XGBoost, SVMs, DCNNs [30]	Robust and scalable anomaly detection for diverse IoT environments	Data heterogeneity, computational efficiency

## Methodology

**Fig. 1:**
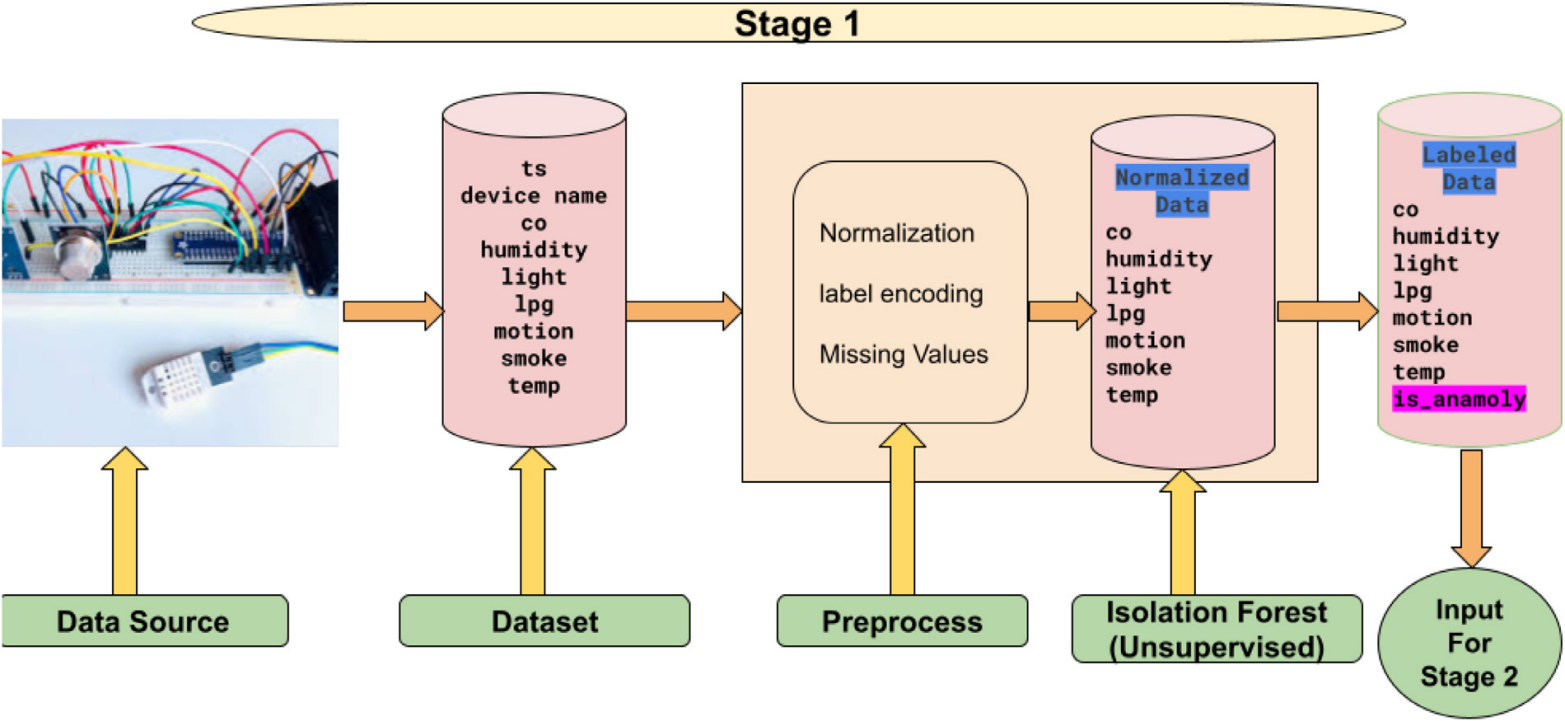
Stage 1 of proposed flow. (Unsupervised learning).

The proposed methodology works in two stages. The Fig. 1 represents Stage 1 of an anomaly detection workflow using environmental sensor data. It begins with the Data Source, a hardware setup capturing sensor data such as ts (timestamp), device name, co, humidity, light, lpg, motion, smoke, and temp. The data flows into a Dataset stage for storage. In the Preprocessing stage, data undergoes normalization, label encoding, and handling of missing values. The processed data is fed into an Isolation Forest (Unsupervised) model, which identifies anomalies and appends an is_anomaly label. The labeled dataset is then prepared as input for Stage 2.

Isolation Forest is an unsupervised anomaly detection algorithm designed to identify anomalies based on how easily a data point can be isolated. It works by randomly selecting features (e.g., co, humidity, etc.) and creating random split points within the range of these features. The algorithm builds a structure of tree using recursive partitioning, which is to split the dataset into smaller subset. For each point, the path length which equates to the number of splits needed to separate a data point — is calculated. Shorter path lengths are generally observed because anomalies are typically isolated more quickly. In the anomaly score, using the average path length across different trees, shorter paths are a better indicator of an anomaly.

This approach is very advantageous to apply on the environmental sensor dataset, primarily because it handles efficiently high dimensional data given without any distributional assumptions about data, i.e., it is robust to handling diverse and complex sensor readings. Isolation Forest requires no labeled data since it fits with this the nature of this study where sensor data begins without assigned anomaly labels. It is also able to detect rare and extreme data points to distinguish anomaly due to sensor faults or unusual environmental conditions. Scalability for real time applications is also ensured by the efficiency of the algorithm's computational part.

*Algorithm for Stage 1:*

**Table:** 

Input: Sensor data: $D=\{\left(x_{1},x_{2},\ldots ,x_{n}\right)\}$, where each $x_{i}$ represents a sensor reading (e.g., co, humidity, etc.).
Output: Labeled dataset: $D^{\prime }=\left\{\left(x_{1},x_{2},\ldots ,x_{n},y\right)\right\}$, where y∈{0,1}(0= Normal, 1 = Anomalous).
Steps:
1. Load Dataset: D← Read data from source (CSV or database)
2. Preprocessing:
• Normalization: Scale each feature $x_{i}$ to the range [0,1] :
$x_{i}^{\prime }=\frac{x_{i}-min\left(x_{i}\right)}{max\left(x_{i}\right)-min\left(x_{i}\right)}$
• Handle Missing Values: Replace $x_{i}$ where $x_{i}=NaN$ with the mean or median of the feature.
• Encoding: Convert categorical data into numerical values (if applicable).
3. Train Isolation Forest: Fit an Isolation Forest model $f_{Iso\;}$ using :
$f_{Iso\;}\left(D\right)\to \;Anomaly\;scores\;for\;each\;x_{i}$
4. Predict Anomalies: Assign anomaly labels y based on a threshold :
y={1,ifanomalyscore>τ0,otherwise
5. Create Labeled Dataset: Combine normalized data D with labels y to form $D^{\prime }$.
6. Output Labeled Dataset: Save $D^{\prime }$ as input for Stage 2.

**Fig. 2:**
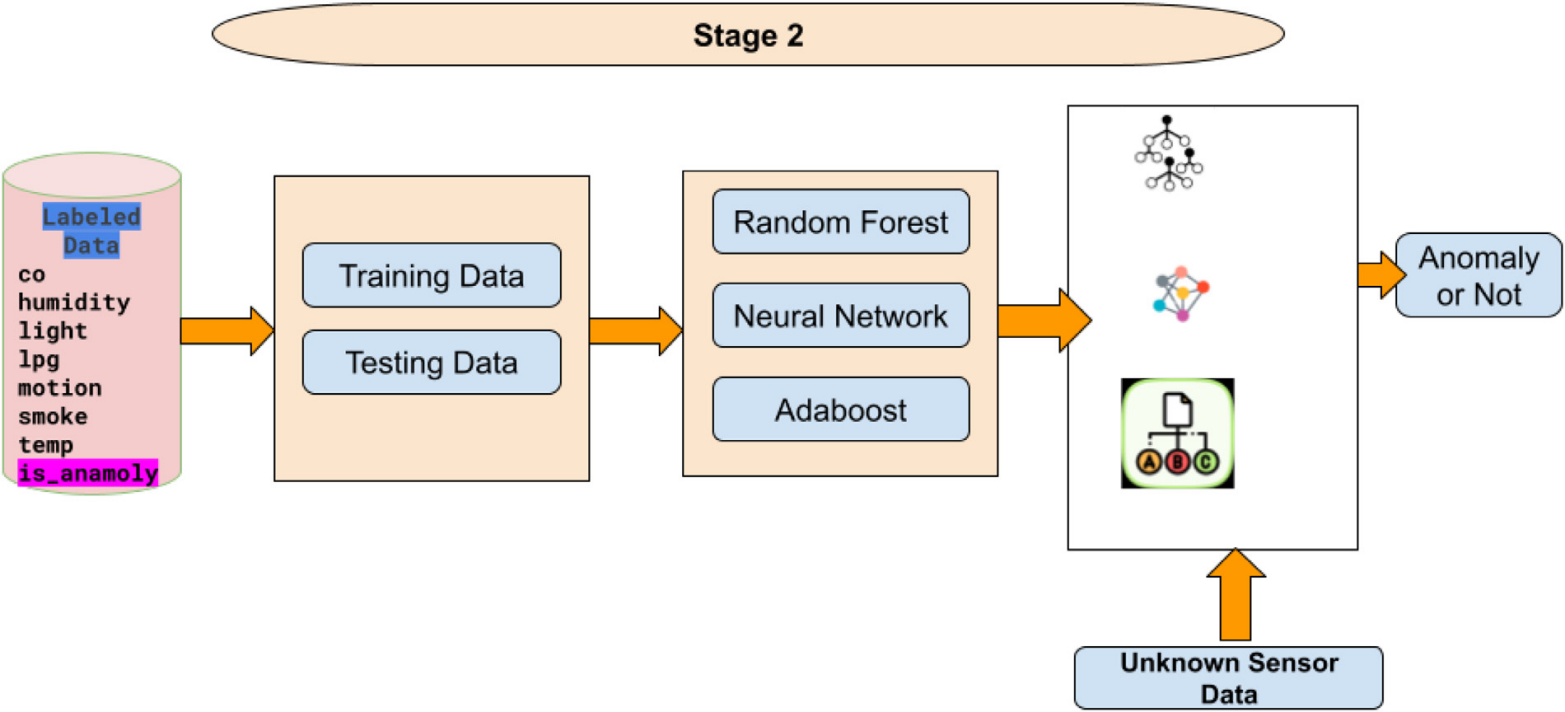
Stage 2 of proposed methodology (Supervised learning).

Stage 2 of a supervised machine learning pipeline for anomaly detection is shown in the Fig. 2. This part starts with the labeled dataset, with sensor reading and is_anomaly label generated in Stage 1. First the dataset gets split into training and testing subsets. The labeled training data is used to train three supervised learning models Random Forest, Neural Network, and AdaBoost. These models are then used to classify unknown sensor data as either anomalous or normal. The pipeline highlights the use of predictive models to determine whether incoming sensor readings indicate an anomaly, facilitating real-time monitoring and fault detection in environmental sensor systems.

*Algorithm for Stage 2:*

**Table:** 

Input: Labeled dataset: $D^{\prime }=\left\{\left(x_{1},x_{2},\ldots ,x_{n},y\right)\right\}$, where y∈{0,1}. New sensor data: $D_{new\;}=\left\{\left(x_{1}^{\prime },x_{2}^{\prime },\ldots ,x_{n}^{\prime }\right)\right\}$.
Output: Prediction $y_{pred\;}\in \left\{0,1\right\}$ for $D_{new\;}$ (Anomaly or Not). Steps:
1. Split Dataset: Divide $D^{\prime }$ into:
• Training set $D_{train\;}$
• Testing set $D_{test\;}$
2. Train Supervised Models:
• Random Forest:
$f_{RF}\leftarrow Train\left(D_{train\;}\right)$
• Neural Network:
$f_{NN}\leftarrow Train\left(D_{train\;}\right)$
• AdaBoost:
$f_{AB}\leftarrow Train\left(D_{train\;}\right)$
3. Evaluate Models: For each model :
• Predict on $D_{test\;}$ :
$y_{pred\;}=f\left(D_{test\;}\right)$
• Compute performance metrics (Accuracy, Precision, Recall, F1 Score).
4. Predict Anomalies for New Data: For $D_{new\;}$, apply each trained model f :
$\;y_{new\;,RF}=f_{RF}\left(D_{new\;}\right)\;\;y_{new\;},NN=f_{NN}\left(D_{new\;}\right)\;\;y_{new\;,AB}=f_{AB}\left(D_{new\;}\right)$
5. Output Predictions: Combine model predictions for $D_{new\;}$ and classify as:
$y_{final\;}=\;majority\;vote\;of\;\left(y_{new\;,RF},y_{new\;},NN,y_{new\;},AB\right)$

Random Forest is an ensemble learning algorithm that builds multiple decision trees during training and merges their outputs (via majority voting or averaging) to improve classification or regression accuracy. Each tree is trained on a random subset of data and features, which reduces overfitting and improves generalization. The randomness in feature selection and data sampling ensures diverse predictions among trees, making the model robust to noise and data imbalance. Random Forest is effective for the environmental sensor dataset as it can handle high-dimensional data and capture non-linear relationships among features like co, humidity, lpg, etc. Its robustness to noise and ability to rank feature importance make it particularly useful for identifying anomalies in new sensor data.

Neural Networks consist of interconnected layers of nodes (neurons) that process input data through weighted connections. In a Multi-Layer Perceptron (MLP), there are three main types of layers: input, hidden, and output. The network uses non-linear activation functions sigmoid and backpropagation to iteratively adjust weights and minimize the loss function. Neural Networks are highly flexible and can model complex, non-linear patterns in data. Neural Networks are well-suited for capturing complex and non-linear interactions in the sensor data. They can effectively differentiate between subtle patterns that may indicate anomalies. This is especially useful in cases where anomalies arise from complex dependencies among multiple features (e.g., combinations of high humidity and smoke levels).

AdaBoost (Adaptive Boosting) is an ensemble method that combines multiple weak classifiers (often decision stumps) to create a strong classifier. It works iteratively, adjusting the weights of incorrectly classified samples to focus more on difficult cases. Each subsequent model attempts to correct the mistakes of its predecessor. The final output is a weighted vote of all classifiers. AdaBoost's iterative focus on difficult-to-classify samples makes it effective for detecting anomalies in sensor data, especially when some anomaly patterns are subtle or rare. It ensures that even challenging data points are accurately classified, contributing to higher overall model performance.

## Method validation

### Dataset description

For this research, we used the dataset downloaded from Kaggle's Environmental Sensor Telemetry Data which carries 405,184 sensor readings resulting from multiple IoT devices in various environmental conditions [31]. An alias of each reading contains parameters like carbon monoxide (CO), humidity, liquid petroleum gas (LPG), smoke, temperature, light (binary), and motion (binary) and a timestamp. It consists of 7 days to perform anomaly detection and classification in with a wealth of data. The dataset did not have any missing values, which is a very good pre-processing and analysis starting point.

### Preprocessing results

**Fig. 3:**
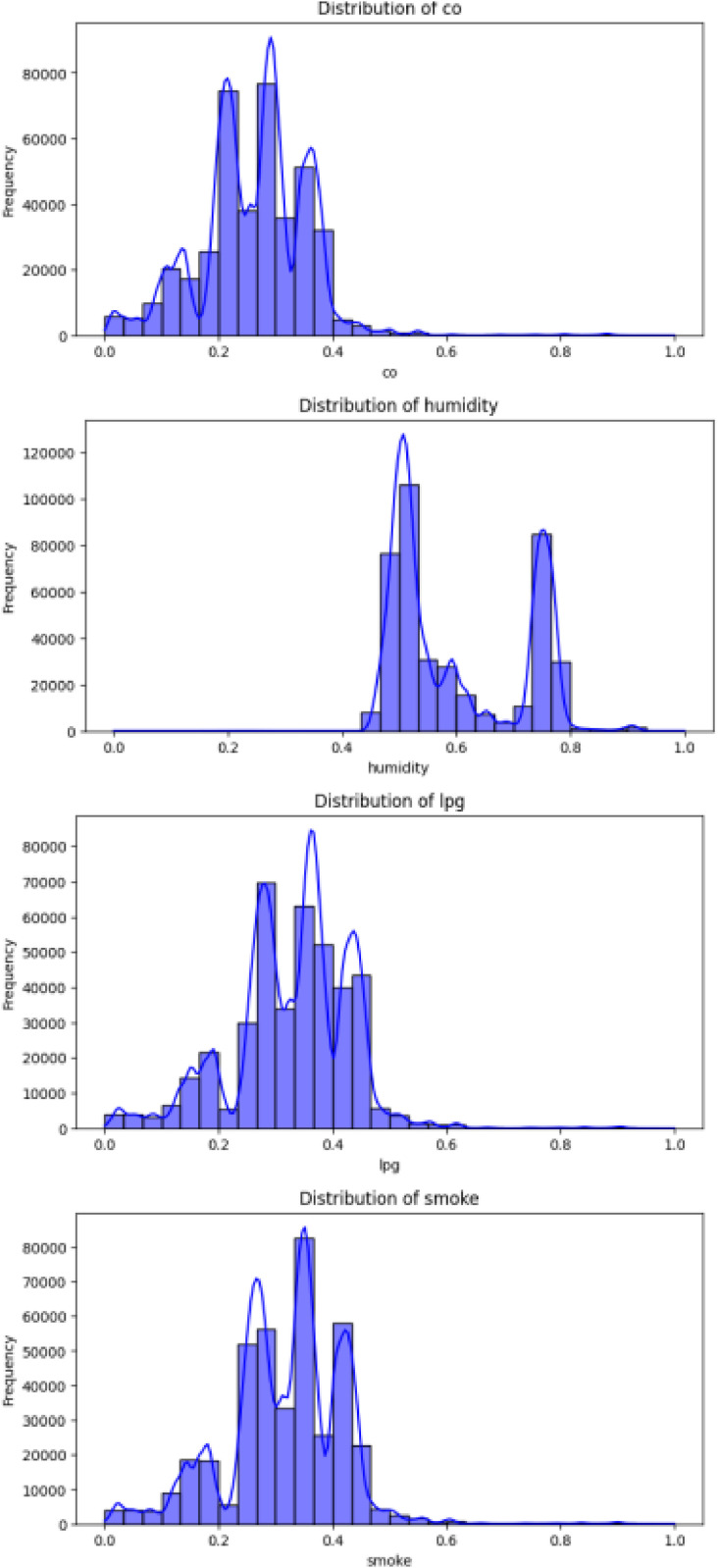
Data distribution.

The dataset was preprocessed to prepare it for anomaly detection and classification tasks:

Continuous features (co, humidity, lpg, smoke, temp) were scaled to a range of 0 to 1 using MinMaxScaler. This ensured all features had equal influence on the models. The distribution of each feature was plotted in Fig. 3, revealing the variation in sensor readings. Features like CO and LPG showed a wider spread, indicating potential outliers or anomalies. After normalization, the dataset was free from scale disparities, ready for anomaly detection.

**Fig. 4:**
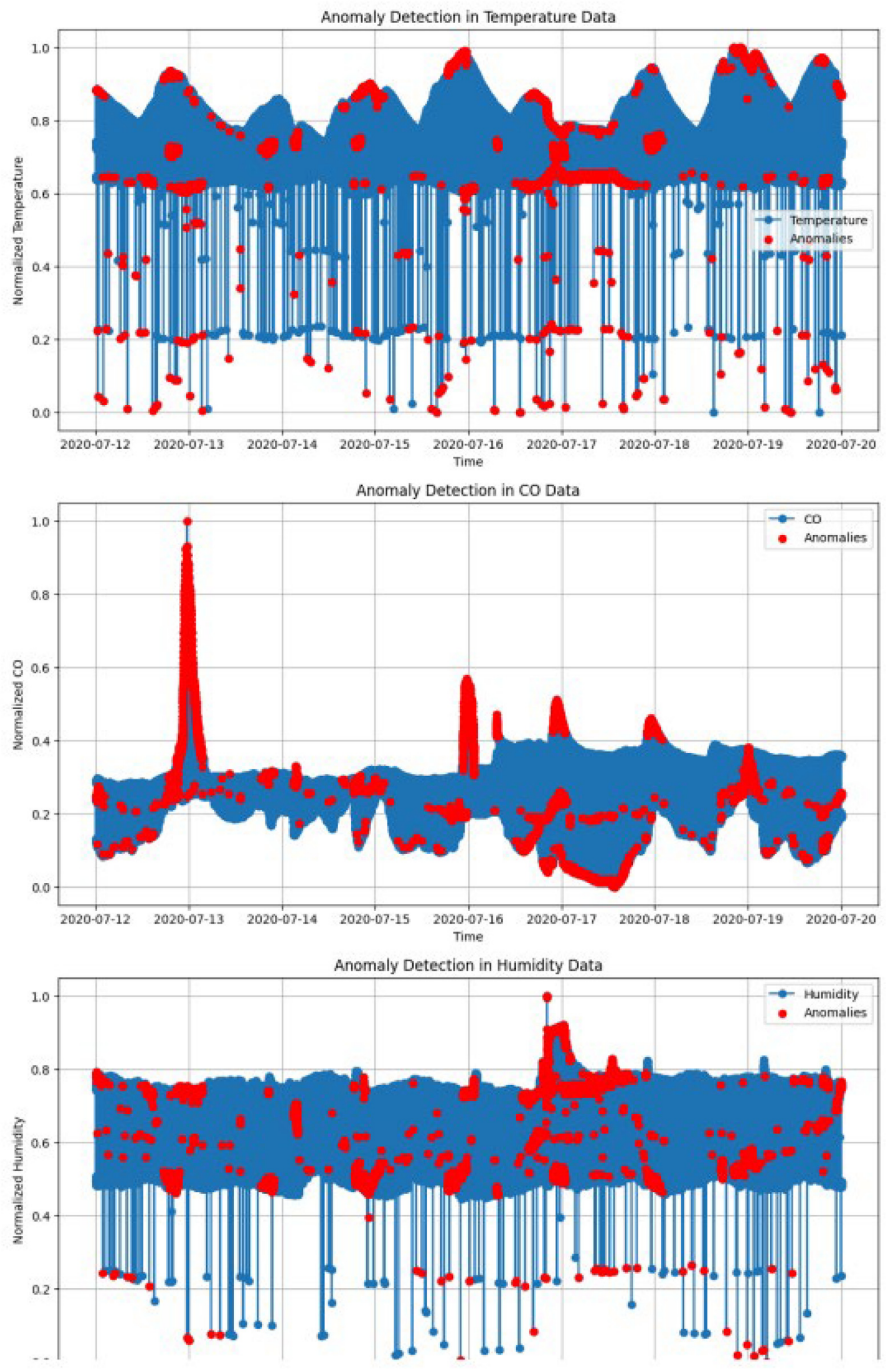
Anomaly detection of sensor reading.

Using the Isolation Forest algorithm, anomalies were detected in the dataset. The algorithm generated an is_anomaly column, marking records as either anomalous or normal. Approximately 10 % of the dataset (40,512 records) were flagged as anomalies. Fig. 4 shows the visual representation of anomaly of few attributes reading.

*Visual Analysis:*

1. Temperature Anomalies:
  ○ Visualized by plotting normalized temperature readings over time, with anomalies highlighted in red.
  ○ Observations: Spikes in anomalies corresponded to unusual temperature fluctuations, potentially indicative of sensor faults or environmental outliers.
2. CO Anomalies:
  ○ Similarly, CO anomalies were highlighted in red, showing unexpected deviations.
  ○ This insight suggests CO sensors might be more prone to anomalies due to their sensitivity.
3. Other Features:
  ○ LPG, humidity, and smoke readings exhibited similar anomaly patterns, aligning with environmental variations or sensor issues.

**Fig. 5:**
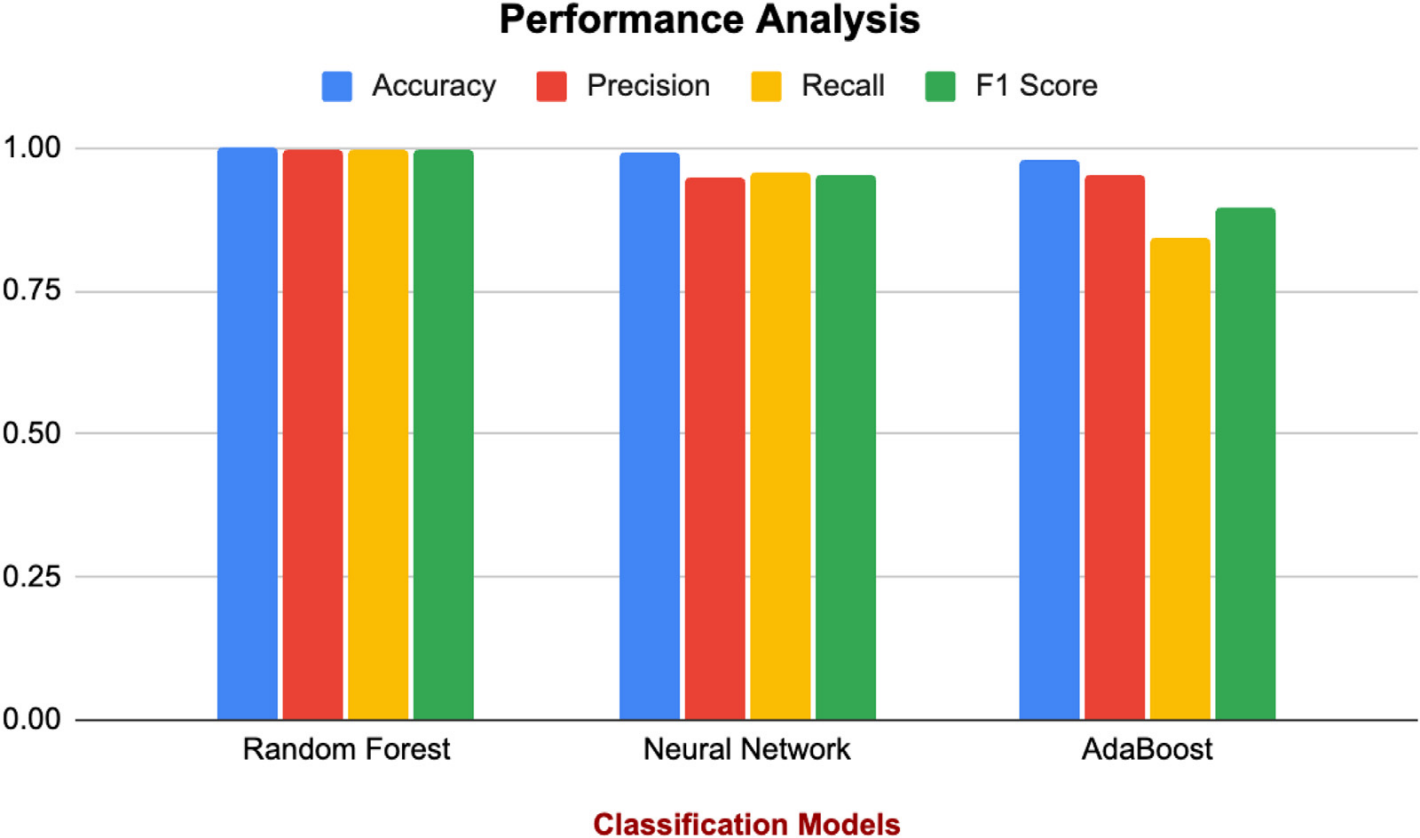
Result analysis.

Supervised learning models (Random Forest, Neural Network, and AdaBoost) were trained using the labeled dataset generated by the Isolation Forest. The dataset was split into training (70 %) and testing (30 %) sets, and performance metrics were evaluated on the test data. Its performance is described in Table 2. And visualized graphically in Fig. 5

**Table 2:** Classification performance.

Model	Accuracy	Precision	Recall	F1 Score
Random Forest	0.9993	0.9975	0.9951	0.9963
Neural Network	0.9905	0.9481	0.9567	0.9524
AdaBoost	0.9804	0.9536	0.8432	0.8950

Random Forest achieved the highest accuracy (99.93 %), indicating its ability to classify nearly all instances correctly. This highlights its robustness and effectiveness in handling complex, high-dimensional sensor data with minimal errors. Neural Network also performed well, achieving an accuracy of 99.05 %. The slight drop compared to Random Forest suggests that Neural Networks may require more fine-tuning to reach optimal performance, especially with diverse and non-linear sensor data patterns. AdaBoost, while slightly behind, achieved a respectable accuracy of 98.04 %. Its iterative focus on difficult-to-classify instances enables it to maintain competitive performance, albeit slightly less reliable compared to Random Forest.

Precision measures the proportion of correctly identified anomalies out of all instances predicted as anomalies. Random Forest achieved near-perfect precision (99.75 %), demonstrating its ability to avoid false positives. This ensures that normal sensor readings are rarely misclassified as anomalies, reducing unnecessary alerts. Neural Network exhibited slightly lower precision (94.81 %), indicating that it may generate more false positives compared to Random Forest. However, this is still acceptable for many applications where capturing all possible anomalies is critical. AdaBoost showed similar precision (95.24 %) to Neural Network, performing reasonably well in reducing false positives despite its lower overall accuracy.

Recall measures the proportion of correctly identified anomalies out of all true anomalies. Random Forest maintained high recall (99.51 %), ensuring that nearly all true anomalies were detected. This makes it highly reliable for environments where missing anomalies could lead to significant risks. Neural Network had slightly higher recall (95.67 %) than its precision, indicating its strength in capturing true anomalies at the cost of some false positives. This is particularly useful in applications where missing anomalies is costlier than generating false alarms. But AdaBoost fell short at a recall of only 84.32 %. Therefore, it implies its capability for leaving a considerable amount of true anomalies untouched, which is not desirable for cases where all anomalies have to be detected.

The highest value of F1 Score (99.63 %) demonstrates that Random Forest is capable of achieving meanwhile anomaly detection and normal classification. Finally, Neural Network scored 95.24 % with an F1 Score which was slightly less reliable than Random Forest, in order to balance precision and recall, albeit successfully. Its low recall earned it an F1 Score of 89.50 %. It will reliably identify anomalies, but its capability to capture much of the true anomalies undermines its overall reliability.

All metrics were found to be extraordinarily performed, and thus, Random Forest is an ideal model for anomaly detection in this study. It is robust against noise, can handle high dimensional data, and has very low hyperparameter tuning requirements, which makes it perfect for use in real time IoT based sensor systems. As far as recall goes, the phenomenal performance of Neural Networks was shown. This indicates that they are able to detect such complex anomaly patterns as not to be detected by simpler models such as Random Forest or AdaBoost. But they need more computation resources and fine tuning, making them hard to apply in resource constrained environments. Results show that AdaBoost achieved high precision and F1 Score while its lower recall might miss out on pertinent anomalies. But its simplicity and ability to focus on exotic instances, while sacrificing exhaustive anomaly detection will serve as a valuable alternative in case computational efficiency is given preference over exhaustive anomaly detection.

As per the result analysis of the methodology, the gold methods for predictive maintenance and anomaly detection include techniques like Random Forests for robustness and handling high-dimensional data, Neural Networks for capturing complex patterns, Isolation Forests for unsupervised anomaly detection, and AdaBoost for focusing on hard-to-classify cases. These methods are widely recognized for their scalability, precision, and adaptability.

Here is a test example

Input: pd.DataFrame({‘co’: [0.5], ‘humidity’: [0.6], ‘lpg’: [0.4], ‘smoke’: [0.3], ‘temp’: [0.7], ‘light’: [1], ‘motion’: [0]})

Output:

Predictions for new data (Random Forest): [True]

Predictions for new data (Neural Network): [True]

Predictions for new data (AdaBoost): [True]

## Discussion

This study bridges a significant gap in anomaly detection by innovatively combining unsupervised learning techniques with supervised models, offering a scalable and actionable framework for real-time predictive maintenance in environmental monitoring systems. Using our robust machine learning pipeline, the research was capable of detecting anomalies and classifying sensor data anomalies. Preprocessing on the dataset involved providing high quality unsupervised ins inputs for the models, and the use of the unsupervised Isolation Forest successfully labelled anomalies. A comparative analysis of its strengths over the other supervised models was done, and found that in this context Random Forest was a better choice. The anomaly visualization helped inform sensor performance and environmental patterns that can aid in maintenance and decision making. The high accuracy and precision of the models confirm their suitability to be deployed in real time in IoT sensor systems. Further work could look at advanced deep learning models to achieve further enhancement in predictive abilities. Some of the comparison results are produced as shown in Table 3, for which unsupervised is applied and how it addresses the unlabeled class problem.

**Table 3:** Comparative analysis.

ML Model	Dataset	Accuracy
CNN, GRU, Bi-LSTM (DeepDetect)	NSL-KDD	99.31 % (multi-class), 99.12 % (binary)
One-Class SVM	NASA bearing data, prototype and real industrial machine	>85 % recall
Multi-channel CNN, LSTM	Gulf of Mexico buoy dataset, Hurricane Katrina data	10 % improvement over baseline methods
Hierarchical Temporal Memory (HTM)	Rome taxi GPS (injected faults)	95.15 %
Gaussian Mixture Model (GMM)	IoT-based temperature monitoring	Precision: 1.00, Recall: 0.57, F1: 0.73
HMM, SVM	PhysioNet 2017	98.66 %
Bayesian optimization with ML ensemble	IoTID20, IoT-23	F1 score increased by 10–30 %
Naive Bayes, SVM, Decision Trees, CNN	IoT-23	93 % (Decision Trees)
Random Forest, kNN, ANN	High-dimensional environmental sensor data	0.98 F1 (ANN)
Logistic Regression, Naive Bayes, Random Forest, AdaBoost, SVM	DAD (MQTT-IoT traffic dataset)	99.98 % (Random Forest, AdaBoost)
XGBoost, SVM, DCNN	IoT-23, NSL-KDD, TON_IoT	99.98 % (XGBoost)
Proposed Method( Isolation)	Environmental Sensor Telemetry Data	99.93 (RandomForest)

Advantages of using a two-step machine learning approach include enhanced scalability, robustness to high-dimensional data, and the ability to detect diverse anomaly patterns. Disadvantages involve dependency on computational resources, fine-tuning for supervised models, and potential false positives in complex cases.

## Limitations

Not applicable.

## Ethics statements

This research did not involve human participants, animal experiments, or data collected from social media platforms. All data utilized in this study were collected by researchers adhering to the respective ethical guidelines and without violating privacy rights. No additional ethical approval was required for the use of these datasets in our study.

## CRediT authorship contribution statement

**Saiprasad Potharaju:** Conceptualization, Methodology, Software. **Ravi Kumar Tirandasu:** Supervision. **Swapnali N. Tambe:** Data curation, Writing – original draft. **Devyani Bhamare Jadhav:** Visualization, Investigation. **Dudla Anil Kumar:** Software, Validation. **Shanmuk Srinivas Amiripalli:** Writing – review & editing.

## Declaration of competing interest

The authors declare that they have no known competing financial interests or personal relationships that could have appeared to influence the work reported in this paper.

## Data Availability

Data will be made available on request.

## References

[1] Sarker I.H. (2022). Smart city data science: towards data-driven smart cities with open research issues. Internet Things.

[2] Erhan L., Ndubuaku M., Di Mauro M., Song W., Chen M., Fortino G., Bagdasar O., Liotta A. (2021). Smart anomaly detection in sensor systems: a multi-perspective review. Inf. Fusion.

[3] Ismail Z.A. (2023). Implementation of automation system-based model checking for managing imperfect maintenance actions in chemical plant projects. Ind. Manag. Data Syst..

[4] Bertolini M., Mezzogori D., Neroni M., Zammori F. (2021). Machine learning for industrial applications: a comprehensive literature review. Expert. Syst. Appl..

[5] Nizeyimana E., Hanyurwimfura D., Hwang J., Nsenga J., Regassa D. (2023). Prototype of monitoring transportation pollution spikes through the internet of things edge networks. Sensors.

[6] Wu D., Tang J., Yu Z., Gao Y., Zeng Y., Tang D., Liu X. (2024). Pt/Zn-TCPP nanozyme-based flexible immunoassay for dual-mode pressure–temperature monitoring of low-abundance proteins. Anal. Chem..

[7] Yu Z., Qiu C., Huang L., Gao Y., Tang D. (2023). Microelectromechanical microsystems-supported photothermal immunoassay for point-of-care testing of aflatoxin B1 in foodstuff. Anal. Chem..

[8] Rihhadatulaisy Z.H., Irianto K.D. (2024). Designing an automatic room temperature control system for smart homes for the elderly using IoT. Int. J. Softw. Eng. Comput. Sci. (IJSECS).

[9] Cheong C., Li S., Cao Y., Zhang X., Liu D. (2024). False message detection in Internet of Vehicle through machine learning and vehicle consensus. Inf. Process. Manage.

[10] Yu Z., Tang D. (2022). Artificial neural network-assisted wearable flexible sweat patch for drug management in Parkinson's patients based on vacancy-engineered processing of g-C3N4. Anal. Chem..

[11] Deborah R.A., Prabhudas S. (2023). Enhancing predictive maintenance with a hybrid anomaly detection for real-time industry 4.0 systems. Dogo Rangsang Res. J. UGC Care Group I J..

[12] Zulfiqar Z., Malik S.U.R., Moqurrab S.A., Zulfiqar Z., Yaseen U., Srivastava G. (2024). DeepDetect: an innovative hybrid deep learning framework for anomaly detection in IoT networks. J. Comput. Sci..

[13] Morselli F., Bedogni L., Mirani U., Fantoni M., Galasso S. (2021). Anomaly detection and classification in predictive maintenance tasks with zero initial training. Internet Things.

[14] Karadayi Y., Aydin M.N., Ög˘renci A.S. (2020). A hybrid deep learning framework for unsupervised anomaly detection in multivariate spatio-temporal data. Appl. Sci. (Switzerland).

[15] Fernandes M., Corchado J.M., Marreiros G. (2022). Machine learning techniques applied to mechanical fault diagnosis and fault prognosis in the context of real industrial manufacturing use-cases: a systematic literature review. Appl. Intell..

[16] Zidi S., Alaya B., Moulahi T., Al-Shargabi A., Khediri S.el (2024). Fault prediction and recovery using machine learning techniques and the HTM algorithm in vehicular network environment. IEEE Open J. Intell. Transp. Syst..

[17] Chatterjee A., Ahmed B.S. (2022). Internet of Things (Netherlands).

[18] Bezdek J.C., Rajasegarar S., Moshtaghi M., Leckie C., Palaniswami M., Havens T.C. (2021). Anomaly detection in environmental monitoring networks. IEEe Comput. Intell. Mag..

[19] Grace Hannah, D., Sampath Dakshina Murthy, D., Kalnoor, G., Vetriselvan, M., & Nidhya, D. (2023). *Machine learning algorithms for anomaly detection in IoT networks. S13*, 560–565. www.migrationletters.com

[20] Raje V.V., Goel S., Patil S.V., Kokate M.D., Mane D.A., Lavate S. (2023). Realtime anomaly detection in healthcare IoT: a machine learning-driven security framework. J. Electr. Syst..

[21] Chevtchenko S.F., Rocha E.D.S., Santos M.C.M.d., Mota R.L., Vieira D.M., de Andrade E.C., de Araujo D.R.B. (2023). Anomaly detection in industrial machinery using IoT devices and machine learning: a systematic mapping. IEEe Access..

[22] Lai T., Farid F., Bello A., Sabrina F. (2024). Ensemble learning based anomaly detection for IoT cybersecurity via Bayesian hyperparameters sensitivity analysis. Cybersecur. (Singap).

[23] B.M. Elzaghmouri, Securing industrial iot environments through machine learning-based anomaly detection in the age of pervasive connectivity, Available at SSRN 4625111, 2023. doi:10.2139/ssrn.4625111.

[24] Ullo S.L., Sinha G.R. (2020). Sensors (Switzerland).

[25] Yang M., Zhang J. (2023). Data anomaly detection in the internet of things: a review of current trends and research challenges. Int. J. Adv. Comput. Sci. Appl..

[26] Russo S., Lürig M., Hao W., Matthews B., Villez K. (2020). Active learning for anomaly detection in environmental data. Environ. Model. Softw..

[27] Shiva K., Etikani P., Bhaskar V.V.S.R., Mittal A., Dave A., Thakkar D., Kanchetti D., Munirathnam R. (2024). Anomaly detection in sensor data with machine learning: predictive maintenance for industrial systems. J. Electr. Syst..

[28] Liu Y., Pang Z., Karlsson M., Gong S. (2020). Anomaly detection based on machine learning in IoT-based vertical plant wall for indoor climate control. Build. Environ..

[29] Vigoya L., Fernandez D., Carneiro V., Nóvoa F.J. (2021). IoT dataset validation using machine learning techniques for traffic anomaly detection. Electronics (Switzerland).

[30] Balega M., Farag W., Wu X.W., Ezekiel S., Good Z. (2024). Enhancing IoT security: optimizing anomaly detection through machine learning. Electronics (Switzerland).

[31] Stafford G. https://www.kaggle.com/datasets/garystafford/environmental-sensor-data-132k.

